# Effect of specific and sensitive interventions for the social protection of people affected by tuberculosis: a meta-analysis

**DOI:** 10.1186/s13690-025-01659-4

**Published:** 2025-10-13

**Authors:** Melisane Regina Lima Ferreira, Jesús David Cortés Gil, Rubia Laine de Paula Andrade, Débora de Almeida Soares, Aline Aparecida Monroe, Inês Fronteira

**Affiliations:** 1 University of Sao Paulo at Ribeirao Preto College of Nursing, Ribeirao Preto, Sao Paulo, Brazil; 2 Public Health Research Centre, Comprehensive Health Research Center, NOVA National School of Public Health, NOVA University Lisbon, Lisbon, Portugal; 3 Global Health and Tropical Medicine, Institute of Hygiene and Tropical Medicine, NOVA University Lisbon, Lisbon, Portugal

**Keywords:** Tuberculosis, Public policy, Health policy, Social welfare, Social determinants of health, Human rights

## Abstract

**Background:**

Tuberculosis (TB)-specific interventions directly benefit people affected by the disease and their families, as they are integrated into existing TB treatment programs. Conversely, TB-sensitive interventions form part of a broader social protection framework aimed at the general population. We conducted a meta-analysis of specific and sensitive interventions of social protection of people affected by TB during treatment.

**Methods:**

Studies that used Odds Ratio (OR) and/or Relative Risk (RR) were included. The magnitude of effects was categorized by population and intervention. Heterogeneity was assessed using I^2^ statistics, and analyses were conducted in Stata version 15.0. A network funnel plot was employed to check for publication bias. The methodological quality of studies was evaluated following The Joanna Briggs Institute recommendations.

**Results:**

Thirteen articles were included, covering 82,602 participants. Interventions primarily involved conditional cash transfers for people affected by TB. The results demonstrated that both TB-specific and TB-sensitive interventions had positive effects on treatment success, with an effect estimate of 2.16 (95% CI: 1.52–3.08) for specific interventions and 1.15 (95% CI: 1.07–1.23) for sensitive interventions.

**Conclusions:**

The findings of this meta-analysis contribute to understand the positive impacts of social interventions during TB treatment. These results have important implications for the management of health and social services. They underscore the need to integrate such interventions into TB control programs and to influence public policies in settings where the disease remains a major health issue, intertwined with cycles of poverty and social exclusion.

**Supplementary Information:**

The online version contains supplementary material available at 10.1186/s13690-025-01659-4.

**Table:** 

Text box 1. Contributions to the literature	
•The impact of interventions designed to offer social protection for people affected by TB remains not well understood. •By recognizing TB as not only a medical but also a social issue, the study emphasizes the need to address underlying social determinants of health in TB prevention and treatment efforts. •The results suggest that integrating TB-specific and TB-sensitive interventions into TB control programs can improve treatment success rates. •Given the association between TB and poverty, the incorporation of social protection interventions into TB control programs could contribute to breaking the cycle of poverty and social exclusion perpetuated by the disease.	

## Introduction

Despite the efforts made in recent decades towards preventing tuberculosis (TB) and caring for affected people and their families, envisaging its elimination as a global public health issue, the disease is still highly incident, especially in countries characterized by high income disparity, poverty, and social inequalities. These inequalities encompass limited access to education, employment opportunities, adequate housing, and quality healthcare [1, 2].

Health, as a fundamental human right, is intrinsically linked with access to social protection. A human rights-based approach to TB aligns not only with the goals outlined in The End TB Strategy for TB elimination but also with broader ethical values such as equity, inclusion, participation, and social justice [3].

A review of the literature predominantly identifies TB-specific measures and strategies aimed at providing social protection as a right for people affected by TB. These interventions have the potential to improve the nutritional status and overall quality of life for those undergoing treatment, reduce the catastrophic costs associated with TB, broaden access to healthcare services and interventions, enhance adherence to TB treatment protocols, and ultimately increase TB cure rates [4].

TB-specific interventions directly benefit people affected by the disease and their families, as they are integrated into existing TB treatment programs, for example, food vouchers with a monetary value. Conversely, TB-sensitive interventions are part of broader social protection frameworks aimed at the general population. One example is conditional cash transfers to groups and/or individuals at high risk of TB, which can influence TB outcomes by strengthening economic resilience and alleviating poverty [5].

Therefore, it is imperative to understand how the success of these two types of interventions impact TB as a public health problem. Such understanding can facilitate the incorporation, adaptation, implementation, or transfer of social and health public policies into national TB control programs, while considering local contextual specificities. Consequently, the objective of this meta-analysis was to examine the effects of TB-specific and TB-sensitive interventions enforced during TB treatment to provide social protection for people affected by the disease.

## Methods

This meta-analysis was conducted based on a scoping review published in November 2023 [4] and registered on the Open Science Framework Registries (OSFREGISTRIES) platform (10.17605/OSF.IO/WX3KM). We rigorously followed the methodology outlined in this prior study, as well as the recommendations of the Preferred Reporting Items for Systematic Reviews and Meta-Analyses - Extension for Scoping Reviews (PRISMA-ScR) [6].

To include potential new studies that presented interventions aimed at social protection for people affected by TB, we updated the searches in December 2023 of the following databases: Scopus, Web of Science, Medical Literature Analysis and Retrieval System Online (MEDLINE), *Literatura Latino-Americana e do Caribe em Ciências da Saúde* (LILACS), Embase^®^, and Cumulative Index to Nursing and Allied Health Literature (CINAHL). The search terms used in this updated search are available as a supplementary file ensuring accessibility for readers who wish to replicate or explore the methodology further (See in Additional file 1).

For this meta-analysis, we only included studies that presented TB-specific interventions or TB-sensitive interventions and used, as measure of effect (success of the intervention regard) the Odds Ratio (OR) and/or Relative Risk (RR), aiming to analyze the success of these interventions in ensuring social protection for people affected by TB. In the previously published scoping review [4], 63 articles were identified as relevant for inclusion. However, for the meta-analysis, we selected only studies that reported measures of effect, specifically “OR” or “RR”, resulting in the inclusion of 12 articles.

Comparators included populations not exposed to the interventions, or those receiving standard care or alternative interventions, as described in each included study. The exposures evaluated were strategies aimed at providing social protection (e.g., cash transfers or nutritional aid). The outcomes assessed were primarily related to TB treatment success, programmatic treatment completion, or initiation of TB preventative measures. We sought to identify and analyze publications that reported these strategies implemented by policies, programs, and/or governmental agreements in any context.

The screening process, including title and abstract review, was conducted independently and in a blinded manner by two authors to ensure objectivity and reduce bias. Data extraction was carried out by a single author using a standardized form, and checked by another one to ensure accuracy and consistency.

The magnitude of effects was classified by population and interventions. The standard error of each study was calculated from the reported confidence intervals (CI) along with OR and RR effect measures. Heterogeneity was assessed using the I^2^ statistic. A random-effects model was employed for the analysis (statistical significance set at *p* < 0.05 for RRs and ORs). This model was chosen due to the expected natural heterogeneity in the included studies, as limited research on this topic suggests that treatment impacts are similar but not identical across populations and interventions.

The random-effects model utilized Der Simonian and Laird weights, which balance the contribution of each study to the pooled estimate by accounting for between-study variability. These weights ensure that each study’s influence on the summary estimate reflects its precision and variability, thus enhancing the robustness of the meta-analysis.

All statistical analyses were conducted using Stata version 15.0 (StataCorp^®^). To check for the presence of bias due to studies with small samples, which may lead to publication bias, a network funnel plot was created to measure the effect size of the type of face and visually inspected using symmetry criteria (See in Additional file2).

A flowchart was prepared to present the process of updating the search in the databases. The methodological quality of the articles included in this meta-analysis was assessed using the checklists recommended by The Joanna Briggs Institute (JBI) [7] for randomized clinical trials, quasi-experimental studies, cohort studies, and analytical cross-sectional studies (See in Additional file3).

Additionally, measures were taken to address publication bias in the meta-analysis, which included utilizing a large number of databases, employing a comprehensive search strategy, and maintaining rigor in the selection and extraction of data.

## Results

**Fig. 1:**
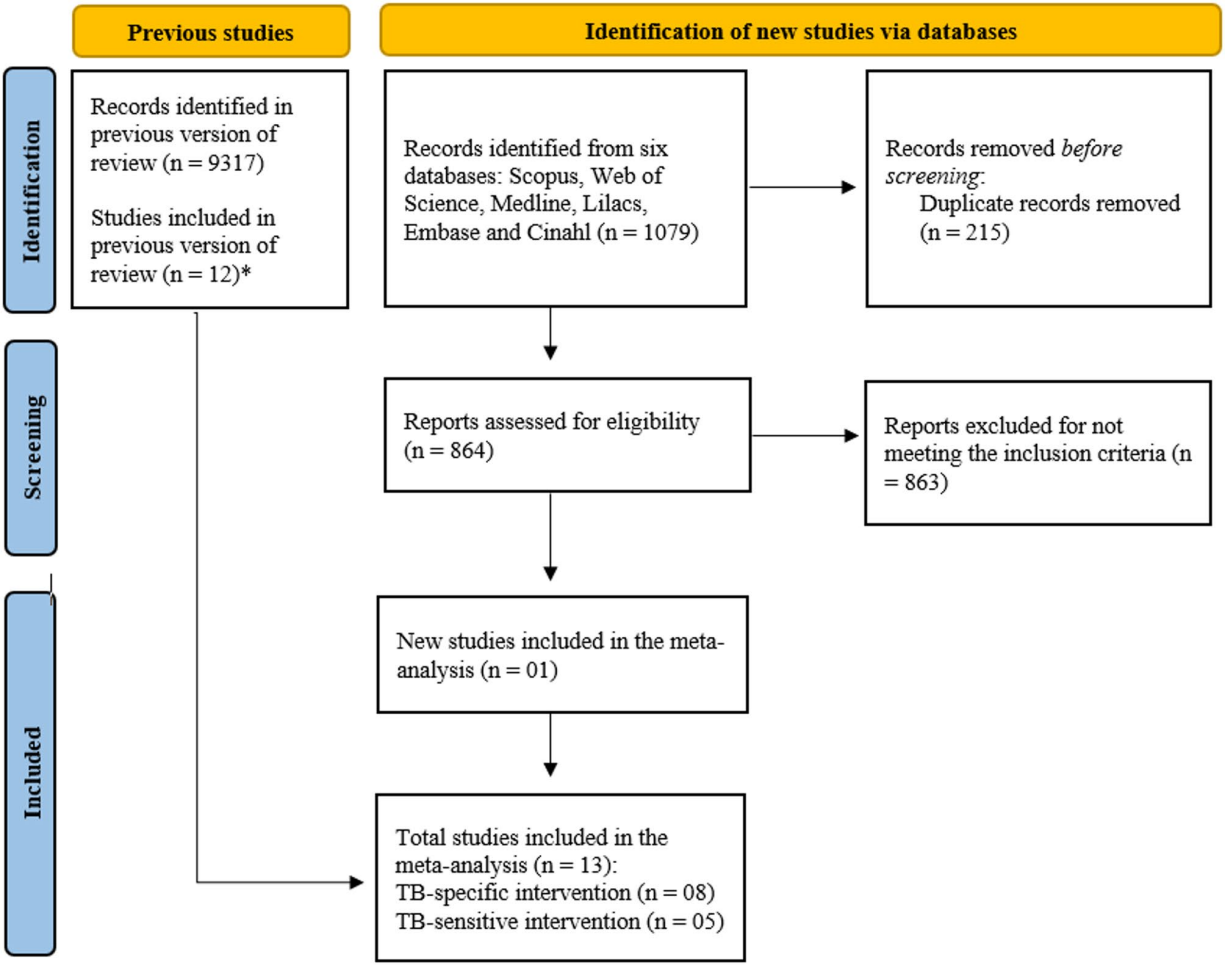
PRISMA Flowchart illustrates updating article searches in databases and included publications in meta-analysis Legend: * In the previously published scoping review, 63 studies were included. However, only 12 of these were able to be incorporated into the meta-analysis. Adapted from [8]

A total of 1,079 publications were identified from the updated searches in the databases. Of these, 215 were excluded due to duplication, 863 were not eligible for full-text reading as they did not meet the inclusion criteria for this meta-analysis, such as interventions outside the scope, or insufficient outcome data. In addition to the studies selected in the initial search, a total of 13 articles were included, comprising eight studies that analyzed TB-specific interventions and five studies that analyzed TB-sensitive interventions (Fig. 1).

The studies included in the meta-analysis exhibited distinct characteristics regarding the implementation context of the intervention, study population, methodological designs, and intervention features. The total sample size was 82,602 participants. The evaluated interventions predominantly encompassed conditional cash transfers to people affected by TB. The description of these characteristics alongside the assessment of methodological quality (AMQ) is presented in Table 1.

**Table 1:** Characteristics of included studies in the meta-analysis (*n* = 13)

Study reference	Country/ income level*	Study design	Sample size	Population characteristics	Intervention	Intervention characteristics	Quality assessment
Kliner et al. 2015 [9]	Swaziland/ Low-income	Experimental Study (Intervention)	1,077	916 people received standard care (control group) and 161 people received support from treatment supporters who were given financial incentives (intervention group)	TB-specific	Financial incentives to family members or community health workers responsible for caring for people with active TB	6/13
Torrens et al. 2016 [10]	Brazil/ Upper-middle-income	Retrospective cohort	7,255	5,788 were people with TB receiving BFP cash transfers during their treatment and 1,467 were people with TB, enrolled in BFP but receiving BFP cash transfers only after the end of treatment	TB-sensitive	Income transfer program	9/11
Wingfield et al. 2017 [11]	Peru/ Upper-middle-income	Non-blinded randomized controlled trial	282	Intervention households (*n* = 135) and control households (*n* = 147)	TB-specific	Conditional cash transfer to people with TB	8/13
Ukwaja et al. 2017 [12]	Nigeria/ Lower-middle-income	Prospective, non-randomized intervention	294	Respectively 173 in the control and intervention periods of 3 months’ duration each	TB-specific	Monthly financial incentive to people with TB	7/9
Durovni et al. 2017 [13]	Brazil/ Upper-middle-income	Retrospective cohort	15,362	13,482 of new TB cases (FHS exposed (*n* = 2,552) and FHS not exposed (*n* = 10,930) 1,880 of retreatment TB cases (FHS exposed (*n* = 338) FHS not exposed (*n* = 1,542)	TB-sensitive	Income transfer program	8/11
Souza et al. 2018 [14]	Brazil/ Upper-middle-income	Ecological study	5,249	-	TB-sensitive	Income transfer program	8/8
Oliosi et al. 2019 [15]	Brazil/ Upper-middle-income	Prospective cohort	1,239	196 individuals were BFP beneficiaries and 1,043 were non-BFP beneficiaries	TB-sensitive	Income transfer program	11/11
Klein et al. 2019 [16]	Argentina/ Upper-middle-income	Prospective cohort	941	CCT registered group: 377 individuals. CCT not registered group: 564 individuals	TB-specific	Conditional cash transfer policy	10/11
Kim et al. 2019 [17]	South Korea/ High-income	Prospective, single-arm, community-based study with historical controls	318	50 in the intervention group (community) and as historical controls, 115 (usual treatment) and 153 (homeless-TB unit).	TB-specific	Housing provision package	5/11
Reis-Santos et al. 2019 [18]	Brazil/ Upper-middle-income	Longitudinal study	25,084	Cash transfer group (beneficiaries of governmental social program): 1,714 individuals	TB-sensitive	Income transfer program	10/11
Modi et al. 2020 [19]	India/ Lower-middle-income	Retrospective cohort	1,860	896 individuals were eligible for the scheme. 394 individuals were not eligible, and 140 individuals had unknown eligibility due to missing information	TB-specific	Financial support for monthly medical care	2/11**
Reis-Santos et al. 2022 [20]	Brazil/ Upper-middle-income	Cluster-randomized controlled trial	774	231 individuals received food vouchers during treatment and 543 individuals received standard treatment	TB-specific	Food voucher (monetary value)	8/13
Sahan et al. 2023 [21]	Uganda/ Low-income	Retrospective cohort	22,867	Received financial social support: 5,033 individuals. Did not receive financial social support: 17,834 individuals.	TB-specific	One-time unconditional cash transfer	5/11

Legend: *The classification of “Country Income Level” was based on the most recent World Bank data (2024), corresponding to the income level of the country where each study was conducted. **Although we included this study during the database searches, it was decided to exclude it from the meta-analysis due to the lack of specific information on exposures, outcomes, statistical analysis, and strategies to address confounding factors and incomplete follow-up, which hinders a comprehensive assessment of the study’s quality. *Abbreviations: BFP*: *Bolsa Família* Program. *FHS*: Family Health Strategy. *CCT*: Conditional Cash Transfer. *TB*: tuberculosis

Regarding the distribution of interventions by national income level, no consistent pattern of effectiveness was observed across countries. However, all TB-sensitive interventions were implemented in Brazil [10, 13–15], an upper-middle-income country. In contrast, TB-specific interventions were more frequently observed in low- and lower-middle-income countries such as Swaziland [9], Nigeria [12], India [19], and Uganda [21].

Further distinctions were found in the conditionality and institutional origin of the interventions. In Brazil, the BFP required compliance with education and health conditions, such as school attendance, childhood vaccination, and prenatal care [14, 18]. In Argentina, the program was linked to treatment adherence and socioeconomic assessments [16]. Both were part of pre-existing social protection frameworks. Conversely, the intervention in South Korea targeted people experiencing homelessness based on eligibility criteria outlined in national policy [17], and the food voucher program in Brazil was implemented without any behavioral conditions [20]. These two were developed specifically for the respective research studies.

**Fig. 2:**
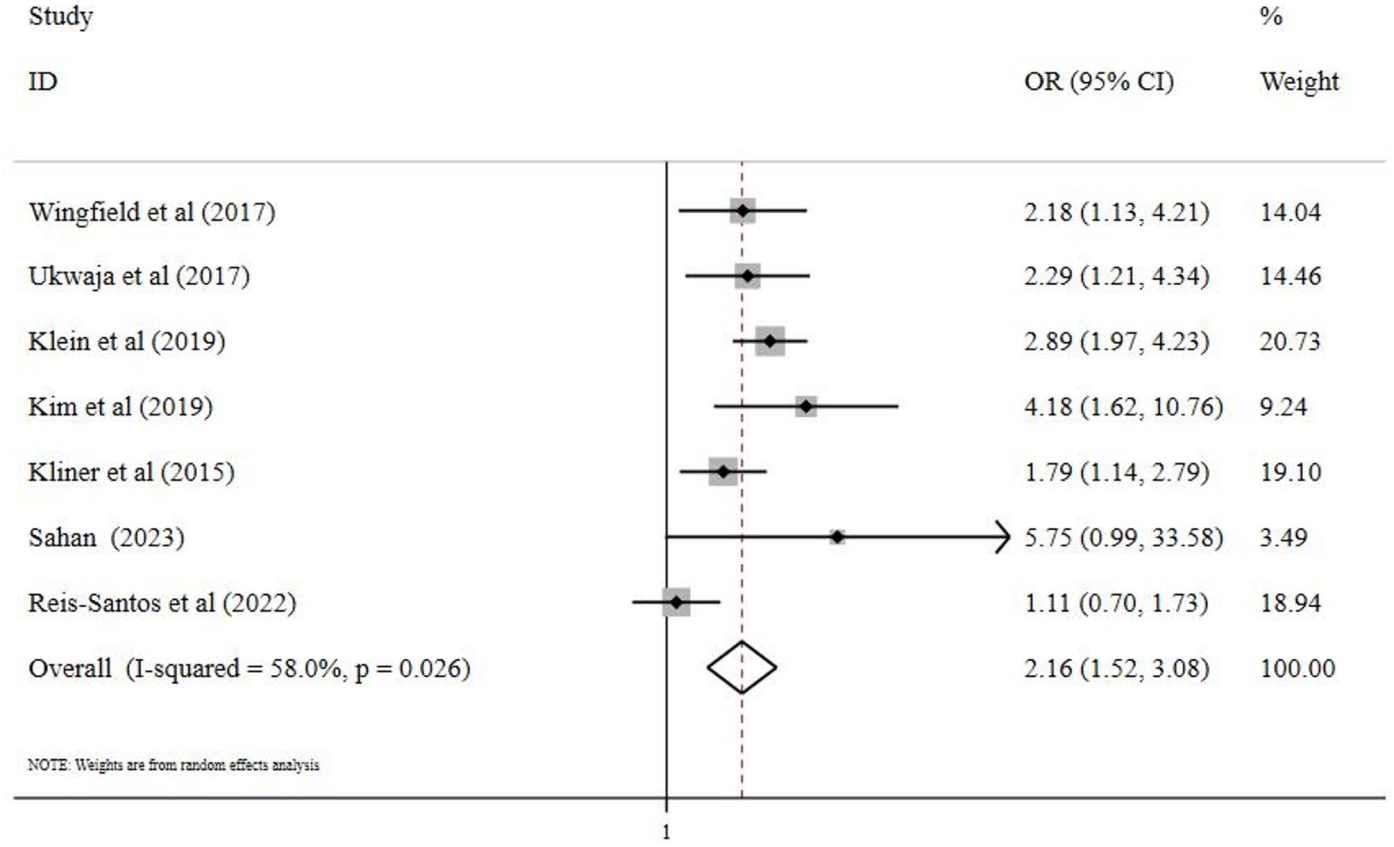
Forest plot compares TB-specific interventions from meta-analysis of seven studies published between 2015–2023 Legend: Squares with bars represent study-specific odds ratios (ORs) with confidence intervals (CIs), and the diamond represents the summary odds ratio estimate. A random-effects model with Der Simonian and Lair weights, equalizing the weight of the studies to the pooled estimate, was used to derive the summary estimate. Assessment of heterogeneity: I^2^ = 58.0%; *p* = 0.026

**Fig. 3:**
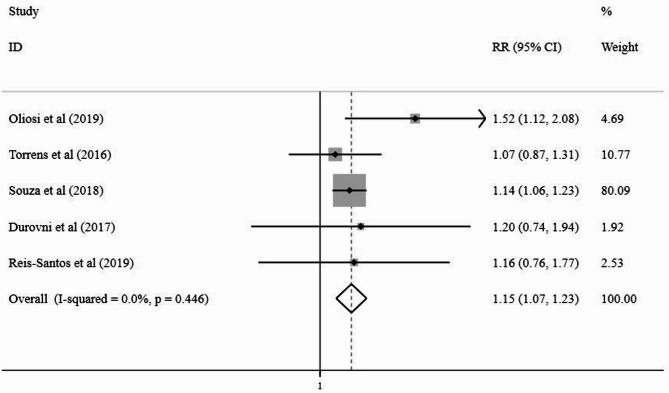
Forest plot compares TB-sensitive interventions from meta-analysis of five studies published between 2016–2019 Legend: Squares with bars represent study-specific odds ratios (RRs) with confidence intervals (CIs), and the diamond represents the summary odds ratio estimate. A random-effects model with Der Simonian and Lair weights, equalizing the weight of the studies to the pooled estimate, was used to derive the summary estimate. Assessment of heterogeneity: I^2^ = 0.0%; *p* = 0.446

Irrespective of the sample size of the included studies, both TB-specific and TB-sensitive interventions showed positive effects on treatment success and TB management. The meta-analysis revealed a significant difference between the studied groups, with an effect estimate (OR) of 2.16 (95% CI: 1.52–3.08) for TB-specific interventions and an effect (RR) of 1.15 (95% CI: 1.07–1.23) for TB-sensitive interventions (Figs. 2 and 3).

The AMQ confirmed the consistency of the main findings, even when different analytical methods were applied. However, some studies exhibited methodological weaknesses, particularly concerning the completeness of follow-up data [10, 13, 16–18, 21], small sample size [17, 21], insufficient strategies to address confounding factors [13, 17, 21], and, in some cases, potencial selection bias due to lack of participant blinding [9, 11]. Heterogeneity was observed among the studies included in the meta-analysis, especially due to their diverse methodological designs and different types of interventions.

## Discussion

The interpretation of the results from this meta-analysis suggests that both TB-specific and TB-sensitive social protection interventions have demonstrated potential effectiveness in achieving successful TB treatment outcomes [9–13, 15–18, 20–21], reducing loss to follow-up [12, 16, 21], TB-related mortality [9, 14], and increasing adherence to drug therapy [16, 20] among people affected by TB. Additionally, one study highlighted the role of these interventions in promoting preventive therapy for household contacts [11].

The observed distribution of intervention types by country income level may reflect structural differences in health systems and the maturity of social protection programs. In upper-middle-income countries like Brazil, TB-sensitive interventions were more commonly embedded in pre-existing social policies. Conversely, low- and lower-middle-income countries such as Swaziland, Nigeria, India, and Uganda more frequently implemented TB-specific interventions, directly linked to treatment programs, likely due to more limited social welfare infrastructure.

These findings have significant implications for the formulation of public and social policies, especially in high TB burden settings with limited resources [9, 12], such as rural and low-income areas [12], and regions with high social inequality [20]. Interventions that promote access to health services, improve food security, and address the social determinants of TB [10, 14] are crucial to support individuals and families affected by the disease. They also contribute to broader objectives, such as reducing health inequities, breaking intergenerational cycles of poverty, and strengthening responses to health emergencies [22].

Furthermore, the results underscore the importance of integrating socioeconomic interventions, such as income transfers (conditional or unconditional) and/or food vouchers, alongside conventional TB treatment, particularly among socially vulnerable populations [11, 13, 16, 20], including individuals experiencing homelessness [17]. A key distinction observed was between interventions implemented within existing social protection programs, such as BFP, and those developed specifically for research purposes. While programs like BFP benefit from institutional continuity and nationwide coverage, their eligibility requirements may exclude highly vulnerable populations, thus limiting their reach and equity.

In contrast, non-conditional interventions developed within research settings, such as the South Korean initiative for people experiencing homelessness [17], respond to specific vulnerabilities but often lack institutional support for sustainability after the study ends. Among the reviewed studies, only the South Korean intervention explicitly considered participants’ conditions after treatment completion. However, none of the interventions assessed long-term outcomes or integration into national policies. These gaps highlight the need for policy strategies that go beyond temporary support and foster sustained social protection aligned with TB elimination efforts, especially in structurally disadvantaged settings.

Taken together, the evidence from this meta-analysis reinforces the importance of sustained investment in universal social and health policies [14]. It may also serve to encourage policymakers to expand inclusive and context-adapted social protection programs to tackle TB and reduce poverty [15], thereby contributing to the achievement of the End TB Strategy and aligning with the Sustainable Development Goals (SDGs 1 and 3).

However, this meta-analysis has limitations that must be acknowledged. The small number of included studies, the heterogeneity of methodological designs and outcomes, and the varying quality of the evidence may have constrained the ability to draw stronger conclusions or offer high-certainty recommendations.

## Conclusions

This meta-analysis advances scientific knowledge by providing insights into the positive effects of TB-specific and TB-sensitive interventions during TB treatment aimed at the social protection of people affected by the disease. These results underscore the importance of comprehensive approaches that consider not only the efficacy of drug treatments but also the socioeconomic contexts of people affected by TB. Ultimately, they have potential implications for the management of healthcare services and social assistance by considering the inclusion of these interventions in TB control programs and may guide health policies and social policies in contexts where TB persists as a serious public health issue, potentially serving as both a cause and consequence of cycles of poverty and social exclusion.

## Electronic supplementary material

Below is the link to the electronic supplementary material.

Supplementary Material 1

Supplementary Material 2

Supplementary Material 3

## Data Availability

No datasets were generated or analysed during the current study.

## Abbreviations

AMQ: Assessment of Methodological Quality
BFP: Bolsa Família Program
CCT: Conditional Cash Transfer
CI: Confidence Intervals
CINAHL: Cumulative Index to Nursing and Allied Health Literature
FHS: Family Health Strategy
LILACS: Latin American and Caribbean Health Sciences Literature
MEDLINE: Medical Literature Analysis and Retrieval System Online
PRISMA-ScR: Preferred Reporting Items for Systematic Reviews and Meta-Analyses - Extension for Scoping Reviews
OR: Odds Ratio
RR: Relative Risk
SDGs: Sustainable Development Goals
TB: Tuberculosis
