# Supplementary material for: Effect of specific and sensitive interventions for the social protection of people affected by tuberculosis: a meta-analysis
Source: Arch Public Health. 2025 Oct 13;83:244. doi: 10.1186/s13690-025-01659-4 (PMC12519751; doi:10.1186/s13690-025-01659-4)
Supplement: Supplementary file 1 — Supplementary Material 1 [file 13690_2025_1659_MOESM1_ESM.docx]

**Additional file 1:** Search strategies for a meta-analysis assessing the impact of TB-specific and TB-sensitive interventions implemented during TB treatment to enhance social protection for people affected by the disease.

| **Databases** | **Search Strategy** |
| --- | --- |
| Scopus | TITLE-ABS-KEY (tuberculosis OR "Koch Disease" OR "Kochs Disease" OR "Mycobacterium tuberculosis Infection" OR "Mycobacterium tuberculosis Infection" OR tuberculoses) AND TITLE-ABS-KEY ("Public Policy" OR "Affirmative Action" OR "Affirmative Actions" OR "Equal Opportunities" OR "Equal Opportunity" OR "Population Policies" OR "Population Policy" OR "Positive Action" OR "Positive Discrimination" OR "Public Policies" OR "Social Policies" OR "Social Policy" OR "Social Protection" OR "Social Welfare" OR "Community Service" OR "Community Services" OR "Public Assistance" OR "Health Policy" OR "Health Policies" OR "Health Public Policies" OR "Health Public Policy" OR "Public Health Policies" OR "Public Health Policy" OR "Welfare Policies" OR "Access to Social Protection" OR "Governmental Social Protection" OR "Social Protection Programmes" OR "Social Protection Interventions" OR "Social Benefits" OR "Income Distribution" OR "Income Generation Program" OR "Financial Support" OR "Income Replacement" OR "Cash Transfer Program" OR "Conditional Cash Transfer" OR "Unconditional Cash Transfer" OR "Bolsa Família Programme" OR "Microfinance Intervention" OR "Socioeconomic Incentives" OR "Economic Support" OR grant OR grants OR "Access to Work" OR "Food Assistance" OR "Food Assistance Program" OR "Food Aid Program" OR "Food Stamp" OR "Food Stamp Program" OR "Supplemental Nutrition Assistance Program" OR "Food Security" OR "Dietary Advocacy" OR "Food Advocacy" OR "Food Rights" OR "Nutritional Advocacy" OR "Nutritional Rights" OR "Right to Adequate Food" OR "Right to Food" OR "Food Insecurity" OR "Food Support" OR "Nutritional Support" OR "Food Baskets" OR "Bus Passes" OR "Transportation Stipends" OR "Social Security" OR "Social Insurance" OR "Government Financing" OR "Government Subsidies" OR "Government Program" OR "Government Sponsored Program" OR "Social Work" OR "Social Service" OR "Social Services" OR "Access to Housing" OR "Housing Program" OR "Human Rights" OR "Collective Human Rights" OR "Right to Decent Housing" OR "Right to Development" OR "Right to Environmental Quality" OR "Right to Housing" OR "Socioeconomic Rights") AND (LIMIT-TO (PUBYEAR , 2023) OR LIMIT-TO (PUBYEAR , 2022) OR LIMIT-TO (PUBYEAR , 2021) OR LIMIT-TO (PUBYEAR , 2020) OR LIMIT-TO (PUBYEAR , 2019) OR LIMIT-TO (PUBYEAR , 2018) OR LIMIT-TO (PUBYEAR , 2017) OR LIMIT-TO (PUBYEAR , 2016) OR LIMIT-TO (PUBYEAR , 2015)) |
| Web of Science | (TS=(Tuberculosis OR "Koch Disease" OR "Kochs Disease" OR "Mycobacterium tuberculosis Infection" OR "Mycobacterium tuberculosis Infection" OR Tuberculoses)) AND TS=("Public Policy" OR "Affirmative Action" OR "Affirmative Actions" OR "Equal Opportunities" OR "Equal Opportunity" OR "Population Policies" OR "Population Policy" OR "Positive Action" OR "Positive Discrimination" OR "Public Policies" OR "Social Policies" OR "Social Policy" OR "Social Protection" OR "Social Welfare" OR "Community Service" OR "Community Services" OR "Public Assistance" OR "Health Policy" OR "Health Policies" OR "Health Public Policies" OR "Health Public Policy" OR "Public Health Policies" OR "Public Health Policy" OR "Welfare Policies" OR "Access to Social Protection" OR "Governmental Social Protection" OR "Social Protection Programmes" OR "Social Protection Interventions" OR "Social Benefits" OR "Income Distribution" OR "Income Generation Program" OR "Financial Support" OR "Income Replacement" OR "Cash Transfer Program" OR "Conditional Cash Transfer" OR "Unconditional Cash Transfer" OR "Bolsa Família Programme" OR "Microfinance Intervention" OR "Socioeconomic Incentives" OR "Economic Support" OR Grant OR Grants OR "Access to Work" OR "Food Assistance" OR "Food Assistance Program" OR "Food Aid Program" OR "Food Stamp" OR "Food Stamp Program" OR "Supplemental Nutrition Assistance Program" OR "Food Security" OR "Dietary Advocacy" OR "Food Advocacy" OR "Food Rights" OR "Nutritional Advocacy" OR "Nutritional Rights" OR "Right to Adequate Food" OR "Right to Food" OR "Food Insecurity" OR "Food Support" OR "Nutritional Support" OR "Food Baskets" OR "Bus Passes" OR "Transportation Stipends" OR "Social Security" OR "Social Insurance" OR "Government Financing" OR "Government Subsidies" OR "Government Program" OR "Government Sponsored Program" OR "Social Work" OR "Social Service" OR "Social Services" OR "Access to Housing" OR "Housing Program" OR "Human Rights" OR "Collective Human Rights" OR "Right to Decent Housing" OR "Right to Development" OR "Right to Environmental Quality" OR "Right to Housing" OR "Socioeconomic Rights") > Filter: year of publication 2015 a 2023 |
| MEDLINE | (("tuberculosis"[All Fields] OR "tuberculosis"[MeSH Terms] OR "tuberculosis"[All Fields] OR "tuberculoses"[All Fields] OR "tuberculosis"[All Fields] OR "Koch Disease"[All Fields] OR ("tuberculosis"[MeSH Terms] OR "tuberculosis"[All Fields] OR ("kochs"[All Fields] AND "disease"[All Fields])) OR "Mycobacterium tuberculosis Infection"[All Fields] OR "Mycobacterium tuberculosis Infection"[All Fields] OR ("tuberculosi"[All Fields] OR "tuberculosis"[MeSH Terms] OR "tuberculosis"[All Fields] OR "tuberculoses"[All Fields] OR "tuberculosis s"[All Fields])) AND ("Public Policy"[All Fields] OR "Affirmative Action"[All Fields] OR "Affirmative Actions"[All Fields] OR "Equal Opportunities"[All Fields] OR "Equal Opportunity"[All Fields] OR "Population Policies"[All Fields] OR "Population Policy"[All Fields] OR "Positive Action"[All Fields] OR "Positive Discrimination"[All Fields] OR "Public Policies"[All Fields] OR "Social Policies"[All Fields] OR "Social Policy"[All Fields] OR "Social Protection"[All Fields] OR "Social Welfare"[All Fields] OR "Community Service"[All Fields] OR "Community Services"[All Fields] OR "Public Assistance"[All Fields] OR "Health Policy"[All Fields] OR "Health Policies"[All Fields] OR "Health Public Policies"[All Fields] OR "Health Public Policy"[All Fields] OR "Public Health Policies"[All Fields] OR "Public Health Policy"[All Fields] OR "Welfare Policies"[All Fields] OR (("access"[All Fields] OR "accessed"[All Fields] OR "accesses"[All Fields] OR "accessibilities"[All Fields] OR "accessibility"[All Fields] OR "accessible"[All Fields] OR "accessing"[All Fields]) AND ("Public Policy"[MeSH Terms] OR ("public"[All Fields] AND "policy"[All Fields]) OR "Public Policy"[All Fields] OR ("social"[All Fields] AND "protection"[All Fields]) OR "Social Protection"[All Fields])) OR (("governmental"[All Fields] OR "governmentality"[All Fields] OR "governmentally"[All Fields]) AND ("Public Policy"[MeSH Terms] OR ("public"[All Fields] AND "policy"[All Fields]) OR "Public Policy"[All Fields] OR ("social"[All Fields] AND "protection"[All Fields]) OR "Social Protection"[All Fields])) OR "Social Protection Programmes"[All Fields] OR "Social Protection Interventions"[All Fields] OR "Social Benefits"[All Fields] OR "Income Distribution"[All Fields] OR "Income Generation Program"[All Fields] OR "Financial Support"[All Fields] OR "Income Replacement"[All Fields] OR "Cash Transfer Program"[All Fields] OR "Conditional Cash Transfer"[All Fields] OR "Unconditional Cash Transfer"[All Fields] OR "Bolsa Familia Programme"[All Fields] OR "Microfinance Intervention"[All Fields] OR "Socioeconomic Incentives"[All Fields] OR "Economic Support"[All Fields] OR ("financing, organized"[MeSH Terms] OR ("financing"[All Fields] AND "organized"[All Fields]) OR "organized financing"[All Fields] OR "grant"[All Fields] OR "grants"[All Fields] OR "granted"[All Fields] OR "granting"[All Fields]) OR ("financing, organized"[MeSH Terms] OR ("financing"[All Fields] AND "organized"[All Fields]) OR "organized financing"[All Fields] OR "grant"[All Fields] OR "grants"[All Fields] OR "granted"[All Fields] OR "granting"[All Fields]) OR "Access to Work"[All Fields] OR "Food Assistance"[All Fields] OR "Food Assistance Program"[All Fields] OR "Food Aid Program"[All Fields] OR "Food Stamp"[All Fields] OR "Food Stamp Program"[All Fields] OR "Supplemental Nutrition Assistance Program"[All Fields] OR "Food Security"[All Fields] OR (("diet"[MeSH Terms] OR "diet"[All Fields] OR "dietary"[All Fields] OR "dietaries"[All Fields]) AND ("advocacies"[All Fields] OR "advocacy"[All Fields] OR "advocacy s"[All Fields])) OR (("food"[MeSH Terms] OR "food"[All Fields]) AND ("advocacies"[All Fields] OR "advocacy"[All Fields] OR "advocacy s"[All Fields])) OR "Food Rights"[All Fields] OR (("nutrition s"[All Fields] OR "nutritional status"[MeSH Terms] OR ("nutritional"[All Fields] AND "status"[All Fields]) OR "nutritional status"[All Fields] OR "nutrition"[All Fields] OR "nutritional sciences"[MeSH Terms] OR ("nutritional"[All Fields] AND "sciences"[All Fields]) OR "nutritional sciences"[All Fields] OR "nutritional"[All Fields] OR "nutritionals"[All Fields] OR "nutritions"[All Fields] OR "nutritive"[All Fields]) AND ("advocacies"[All Fields] OR "advocacy"[All Fields] OR "advocacy s"[All Fields])) OR (("nutrition s"[All Fields] OR "nutritional status"[MeSH Terms] OR ("nutritional"[All Fields] AND "status"[All Fields]) OR "nutritional status"[All Fields] OR "nutrition"[All Fields] OR "nutritional sciences"[MeSH Terms] OR ("nutritional"[All Fields] AND "sciences"[All Fields]) OR "nutritional sciences"[All Fields] OR "nutritional"[All Fields] OR "nutritionals"[All Fields] OR "nutritions"[All Fields] OR "nutritive"[All Fields]) AND ("right"[All Fields] OR "right s"[All Fields] OR "rightful"[All Fields] OR "rights"[All Fields])) OR "Right to Adequate Food"[All Fields] OR "Right to Food"[All Fields] OR "Food Insecurity"[All Fields] OR "Food Support"[All Fields] OR "Nutritional Support"[All Fields] OR "Food Baskets"[All Fields] OR "Bus Passes"[All Fields] OR "transporter"[All Fields] OR "transporters"[All Fields] OR "transportable"[All Fields] OR "transportation"[MeSH Terms] OR "transportation"[All Fields] OR "transportations"[All Fields] OR "transported"[All Fields] OR "transporter s"[All Fields] OR "transporting"[All Fields] OR "transports"[All Fields]) AND ("stipend"[All Fields] OR "stipends"[All Fields])) OR "Social Security"[All Fields] OR "Social Insurance"[All Fields] OR "Government Financing"[All Fields] OR "Government Subsidies"[All Fields] OR "Government Program"[All Fields] OR "Government Sponsored Program"[All Fields] OR "Social Work"[All Fields] OR "Social Service"[All Fields] OR "Social Services"[All Fields] OR (("access"[All Fields] OR "accessed"[All Fields] OR "accesses"[All Fields] OR "accessibilities"[All Fields] OR "accessibility"[All Fields] OR "accessible"[All Fields] OR "accessing"[All Fields]) AND ("home environment"[MeSH Terms] OR ("home"[All Fields] AND "environment"[All Fields]) OR "home environment"[All Fields] OR "house"[All Fields] OR "houses"[All Fields] OR "housing"[MeSH Terms] OR "housing"[All Fields] OR "housed"[All Fields] OR "housings"[All Fields])) OR "Housing Program"[All Fields] OR "Human Rights"[All Fields] OR "Collective Human Rights"[All Fields] OR (("right"[All Fields] OR "right s"[All Fields] OR "rightful"[All Fields] OR "rights"[All Fields]) AND "decent"[All Fields] AND ("home environment"[MeSH Terms] OR ("home"[All Fields] AND "environment"[All Fields]) OR "home environment"[All Fields] OR "house"[All Fields] OR "houses"[All Fields] OR "housing"[MeSH Terms] OR "housing"[All Fields] OR "housed"[All Fields] OR "housings"[All Fields])) OR (("right"[All Fields] OR "right s"[All Fields] OR "rightful"[All Fields] OR "rights"[All Fields]) AND ("develop"[All Fields] OR "develope"[All Fields] OR "developed"[All Fields] OR "developer"[All Fields] OR "developer s"[All Fields] OR "developers"[All Fields] OR "developing"[All Fields] OR "developments"[All Fields] OR "develops"[All Fields] OR "growth and development"[MeSH Subheading] OR ("growth"[All Fields] AND "development"[All Fields]) OR "growth and development"[All Fields] OR "development"[All Fields])) OR (("right"[All Fields] OR "right s"[All Fields] OR "rightful"[All Fields] OR "rights"[All Fields]) AND ("environ qual annu rep counc environ qual"[Journal] OR ("environmental"[All Fields] AND "quality"[All Fields]) OR "environmental quality"[All Fields])) OR "Right to Housing"[All Fields] OR "Socioeconomic Rights"[All Fields])) AND (2015:2023[pdat]) |
| LILACS | (Tuberculose OR "Infecção por Mycobacterium tuberculosis" OR "Pneumologia Sanitária" OR TB OR Tuberculosis OR "Koch Disease" OR "Kochs Disease" OR "Mycobacterium tuberculosis Infection" OR "Mycobacterium tuberculosis Infections" OR Tuberculoses OR "Infección por Mycobacterium tuberculosis") AND ("Política Pública" OR "Políticas Públicas" OR "Ação Afirmativa" OR "Ações Afirmativas" OR "Discriminação Positiva" OR "Igualdade de Oportunidade" OR "Política Populacional" OR "Política Social" OR "Proteção Social" OR "Seguridade Social" OR "Bem-Estar Social" OR "Assistência Pública" OR "Política de Saúde" OR "Políticas de Saúde" OR "Política Pública de Saúde" OR "Políticas Públicas de Saúde" OR "Política de Assistência à Saúde" OR "Políticas de Saúde Pública" OR "Políticas em Saúde Pública" OR "Políticas de Bem-estar" OR "Acesso à Proteção Social" OR "Proteção Social Governamental" OR "Programas de Proteção Social" OR "Benefícios Sociais" OR "Apoio Financeiro" OR "Ajuda Financeira" OR "Reposição de Renda" OR "Programa de Transferência de Renda" OR "Transferência Condicional de Renda" OR "Transferência Incondicional de Renda" OR "Programa Bolsa Família" OR "Intervenção Microfinanceira" OR "Incentivos Socioeconômicos" OR "Suporte Econômico" OR Subsídio OR Subsídios OR "Acesso ao Trabalho" OR "Assistência Alimentar" OR "Ajuda Alimentar" OR "Auxílio Alimentar" OR "Auxílio Alimentação" OR "Cupons de Alimentação" OR "Cupons de Alimentos" OR "Cupons para a Aquisição de Alimentos" OR "Programa de Ajuda Alimentar" OR "Programa de Assistência Nutricional Complementar" OR "Programa de Assistência Nutricional Suplementar" OR "Programa de Cupons de Alimentação" OR "Programas de Ajuda Alimentar" OR "Programas de Assistência Alimentar" OR "Programas de Assistência Nutricional" OR "Segurança Alimentar" OR "Direito à Alimentação" OR "Direito à uma Alimentação Adequada" OR "Direitos Nutricionais" OR "Garantia de Alimentos" OR "Segurança Nutricional" OR "Insegurança Alimentar" OR "Apoio Alimentar" OR "Apoio Nutricional" OR "Cesta Básica" OR "Cestas Básicas" OR "Passe de Ônibus" OR "Vale Transporte" OR "Previdência Social" OR INSS OR "Instituições de Previdência Social" OR "Instituições de Seguro Social" OR "Ministério da Previdência Social" OR "Seguro Social" OR "Sistemas Previdenciários" OR "Financiamento Governamental" OR "Ajuda Federal" OR Subsídio OR Subsídios OR "Subsídio Governamental" OR "Programas Governamentais" OR "Programas Estatais" OR "Programas Patrocinados pelo Governo" OR "Programas Subsidiados pelo Governo" OR "Programas Subvencionados pelo Governo" OR "Serviço Social" OR "Serviços Sociais" OR "Serviços de Assistência Social" OR "Trabalho Social" OR "Acesso à Habitação" OR "Acesso à Moradia" OR "Programa Habitacional" OR "Direitos Humanos" OR "Direito à Habitação" OR "Direito à Moradia Adequada" OR "Direito à Moradia e Abrigo" OR "Direito à Qualidade Ambiental" OR "Direitos Coletivos" OR "Direitos Humanos Coletivos" OR "Direitos Individuais" OR "Direitos da Pessoa Humana" OR "Direitos Socioeconômicos" OR "Public Policy" OR "Affirmative Action" OR "Affirmative Actions" OR "Equal Opportunities" OR "Equal Opportunity" OR "Population Policies" OR "Population Policy" OR "Positive Action" OR "Positive Discrimination" OR "Public Policies" OR "Social Policies" OR "Social Policy" OR "Social Protection" OR "Social Welfare" OR "Community Service" OR "Community Services" OR "Public Assistance" OR "Health Policy" OR "Health Policies" OR "Health Public Policies" OR "Health Public Policy" OR "Public Health Policies" OR "Public Health Policy" OR "Welfare Policies" OR "Access to Social Protection" OR "Governmental Social Protection" OR "Social Protection Programmes" OR "Social Protection Interventions" OR "Social Benefits" OR "Income Distribution" OR "Income Generation Program" OR "Financial Support" OR "Income Replacement" OR "Cash Transfer Program" OR "Conditional Cash Transfer" OR "Unconditional Cash Transfer" OR "Bolsa Família Programme" OR "Microfinance Intervention" OR "Socioeconomic Incentives" OR "Economic Support" OR Grant OR Grants OR "Access to Work" OR "Food Assistance" OR "Food Assistance Program" OR "Food Aid Program" OR "Food Stamp" OR "Food Stamp Program" OR "Supplemental Nutrition Assistance Program" OR "Food Security" OR "Dietary Advocacy" OR "Food Advocacy" OR "Food Rights" OR "Nutritional Advocacy" OR "Nutritional Rights" OR "Right to Adequate Food" OR "Right to Food" OR "Food Insecurity" OR "Food Support" OR "Nutritional Support" OR "Food Baskets" OR "Bus Passes" OR "Transportation Stipends" OR "Social Security" OR "Social Insurance" OR "Government Financing" OR "Government Subsidies" OR "Government Program" OR "Government Sponsored Program" OR "Social Work" OR "Social Service" OR "Social Services" OR "Access to Housing" OR "Housing Program" OR "Human Rights" OR "Collective Human Rights" OR "Right to Decent Housing" OR "Right to Development" OR "Right to Environmental Quality" OR "Right to Housing" OR "Socioeconomic Rights" OR "Política Pública" OR "Acciones Afirmativas" OR "Acción Afirmativa" OR "Discriminación Positiva" OR "Igualdad de Ocasiones" OR "Igualdad de Oportunidad" OR "Igualdad de Oportunidades" OR "Política Social" OR "Políticas Públicas" OR "Protección Social" OR "Bienestar Social" OR "Asistencia Pública" OR "Política de Salud" OR "Políticas de Salud" OR "Políticas de Salud Pública" OR "Políticas en Salud Pública" OR "Políticas Pública de Salud" OR "Políticas Sanitarias" OR "Políticas de Bienestar" OR "Acceso a Protección Social" OR "Protección Social Gubernamental" OR "Programas de Protección Social" OR "Intervenciones de Protección Social" OR "Beneficios Sociales" OR "Acceso al Trabajo" OR "Apoyo Financiero" OR "Ayuda Financiera" OR "Reemplazo de Ingresos" OR "Programa de Transferencia de Ingresos" OR "Transferencia Condicional de Ingresos" OR "Transferencia Incondicional de Ingresos" OR "Intervención de Microfinanzas" OR "Incentivos Socioeconómicos" OR "Apoyo Económico" OR Subsidio OR Subsidios OR "Asistencia Alimentaria" OR "Ayuda Alimentaria" OR "Cupones de Alimentos" OR "Cupones para Alimentos" OR "Estampillas para Alimentos" OR "Programa Asistencial de Nutrición Suplementaria" OR "Programa Suplementario de Asistencia Nutricional" OR "Programa de Asistencia Nutricional Suplementaria" OR "Programa de Asistencia de Nutrición Complementaria" OR "Programas de Ayuda Alimentaria" OR "Programa de Cupones de Alimentos" OR "Programa de Cupones para Alimentos" OR "Programas de Asistencia Alimentaria" OR "Seguridad Alimentaria" OR "Acceso a la Alimentación" OR "Derecho a la Alimentación" OR "Derecho a la Nutrición" OR "Derecho a una Alimentación Adecuada" OR "Derecho de Alimentos" OR "Derechos Nutricionales" OR "Seguridad Alimentaria y Nutricional" OR "Inseguridad Alimentaria" OR "Apoyo Alimenticio" OR "Apoyo Nutricional" OR "Canasta Básica" OR "Pase de Autobús" OR "Vale de Transporte" OR "Seguridad Social" OR "Instituciones de Seguridad Social" OR "Instituciones de Seguro Social" OR "Previsión Social" OR "Regímenes de Seguridad Social" OR "Regímenes de Seguro Social" OR "Seguro Social" OR "Sistemas de Seguro Social" OR "Financiación Gubernamental" OR "Ayuda Federal" OR "Ayuda Gubernamental" OR "Subsidio Gubernamental" OR "Programas de Gobierno" OR "Programas Patrocinados por el Gobierno" OR "Servicio Social" OR "Prestaciones Complementarias" OR "Servicios de Asistencia Social" OR "Servicios Sociales" OR "Trabajo Social" OR "Acceso a Vivienda" OR "Programa de Vivienda" OR "Derechos Humanos" OR "Derecho Colectivo" OR "Derecho a la Calidad Ambiental" OR "Derecho a la Vivienda" OR "Derecho a una Vivienda Digna" OR "Derecho al Alojamiento" OR "Derechos Socioeconómicos") [Palavras] and 2015 OR 2016 OR 2017 OR 2018 OR 2019 OR 2020 OR 2021 OR 2022 OR 2023 [Ano de publicação] |
| Embase | #1 'tuberculosis'/exp OR tuberculosis OR 'koch disease' OR 'kochs disease' OR 'mycobacterium tuberculosis infection'/exp OR 'mycobacterium tuberculosis infection' OR tuberculoses  #2 'public policy'/exp OR 'public policy' OR 'affirmative action'/exp OR 'affirmative action' OR 'affirmative actions' OR 'equal opportunities' OR 'equal opportunity' OR 'population policies' OR 'population policy'/exp OR 'population policy' OR 'positive action' OR 'positive discrimination' OR 'public policies' OR 'social policies' OR 'social policy'/exp OR 'social policy' OR 'social protection'/exp OR 'social protection' OR 'social welfare'/exp OR 'social welfare' OR 'community service'/exp OR 'community service' OR 'community services' OR 'public assistance'/exp OR 'public assistance' OR 'health policy'/exp OR 'health policy' OR 'health policies' OR 'health public policies' OR 'health public policy' OR 'public health policies' OR 'public health policy'/exp OR 'public health policy' OR 'welfare policies' OR 'access to social protection' OR 'governmental social protection' OR 'social protection programmes' OR 'social protection interventions' OR 'social benefits' OR 'income distribution'/exp OR 'income distribution' OR 'income generation program' OR 'financial support'/exp OR 'financial support' OR 'income replacement' OR 'cash transfer program'/exp OR 'cash transfer program' OR 'conditional cash transfer'/exp OR 'conditional cash transfer' OR 'unconditional cash transfer'/exp OR 'unconditional cash transfer' OR 'bolsa família programme' OR 'microfinance intervention' OR 'socioeconomic incentives' OR 'economic support' OR 'grant'/exp OR grant OR 'grants'/exp OR grants OR 'access to work' OR 'food assistance'/exp OR 'food assistance' OR 'food assistance program'/exp OR 'food assistance program' OR 'food aid program'/exp OR 'food aid program' OR 'food stamp' OR 'food stamp program'/exp OR 'food stamp program' OR 'supplemental nutrition assistance program'/exp OR 'supplemental nutrition assistance program' OR 'food security'/exp OR 'food security' OR 'dietary advocacy' OR 'food advocacy' OR 'food rights' OR 'nutritional advocacy' OR 'nutritional rights' OR 'right to adequate food' OR 'right to food' OR 'food insecurity'/exp OR 'food insecurity' OR 'food support' OR 'nutritional support'/exp OR 'nutritional support' OR 'food baskets' OR 'bus passes' OR 'transportation stipends' OR 'social security'/exp OR 'social security' OR 'social insurance'/exp OR 'social insurance' OR 'government financing'/exp OR 'government financing' OR 'government subsidies' OR 'government program' OR 'government sponsored program' OR 'social work'/exp OR 'social work' OR 'social service'/exp OR 'social service' OR 'social services' OR 'access to housing' OR 'housing program' OR 'human rights'/exp OR 'human rights' OR 'collective human rights' OR 'right to decent housing' OR 'right to development' OR 'right to environmental quality' OR 'right to housing' OR 'socioeconomic rights'  #3: #1 AND #2  #4: #3 AND [embase]/lim NOT ([embase]/lim AND [medline]/lim) AND (2015:py OR 2016:py OR 2017:py OR 2018:py OR 2019:py OR 2020:py OR 2021:py OR 2022:py) |
| CINAHL | (Tuberculosis OR "Koch Disease" OR "Kochs Disease" OR "Mycobacterium tuberculosis Infection" OR "Mycobacterium tuberculosis Infection" OR Tuberculoses) AND ("Public Policy" OR "Affirmative Action" OR "Affirmative Actions" OR "Equal Opportunities" OR "Equal Opportunity" OR "Population Policies" OR "Population Policy" OR "Positive Action" OR "Positive Discrimination" OR "Public Policies" OR "Social Policies" OR "Social Policy" OR "Social Protection" OR "Social Welfare" OR "Community Service" OR "Community Services" OR "Public Assistance" OR "Health Policy" OR "Health Policies" OR "Health Public Policies" OR "Health Public Policy" OR "Public Health Policies" OR "Public Health Policy" OR "Welfare Policies" OR "Access to Social Protection" OR "Governmental Social Protection" OR "Social Protection Programmes" OR "Social Protection Interventions" OR "Social Benefits" OR "Income Distribution" OR "Income Generation Program" OR "Financial Support" OR "Income Replacement" OR "Cash Transfer Program" OR "Conditional Cash Transfer" OR "Unconditional Cash Transfer" OR "Bolsa Família Programme" OR "Microfinance Intervention" OR "Socioeconomic Incentives" OR "Economic Support" OR Grant OR Grants OR "Access to Work" OR "Food Assistance" OR "Food Assistance Program" OR "Food Aid Program" OR "Food Stamp" OR "Food Stamp Program" OR "Supplemental Nutrition Assistance Program" OR "Food Security" OR "Dietary Advocacy" OR "Food Advocacy" OR "Food Rights" OR "Nutritional Advocacy" OR "Nutritional Rights" OR "Right to Adequate Food" OR "Right to Food" OR "Food Insecurity" OR "Food Support" OR "Nutritional Support" OR "Food Baskets" OR "Bus Passes" OR "Transportation Stipends" OR "Social Security" OR "Social Insurance" OR "Government Financing" OR "Government Subsidies" OR "Government Program" OR "Government Sponsored Program" OR "Social Work" OR "Social Service" OR "Social Services" OR "Access to Housing" OR "Housing Program" OR "Human Rights" OR "Collective Human Rights" OR "Right to Decent Housing" OR "Right to Development" OR "Right to Environmental Quality" OR "Right to Housing" OR "Socioeconomic Rights") > Filter: year of publication 2015 a 2023 |
