# Supplementary material for: Effect of specific and sensitive interventions for the social protection of people affected by tuberculosis: a meta-analysis
Source: Arch Public Health. 2025 Oct 13;83:244. doi: 10.1186/s13690-025-01659-4 (PMC12519751; doi:10.1186/s13690-025-01659-4)

**Additional File 2**

A) Funnel plot with pseudo 95% confidence limits – exp (OR)


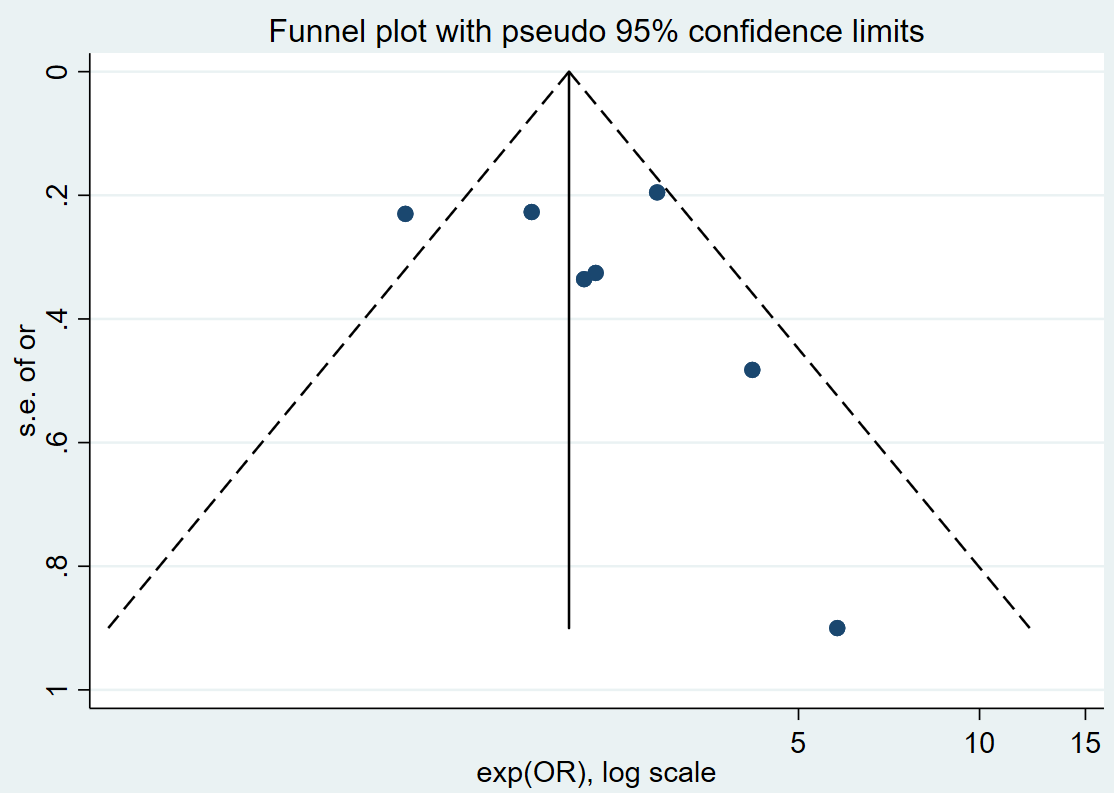


B) Funnel plot with pseudo 95% confidence limits – exp (OR)


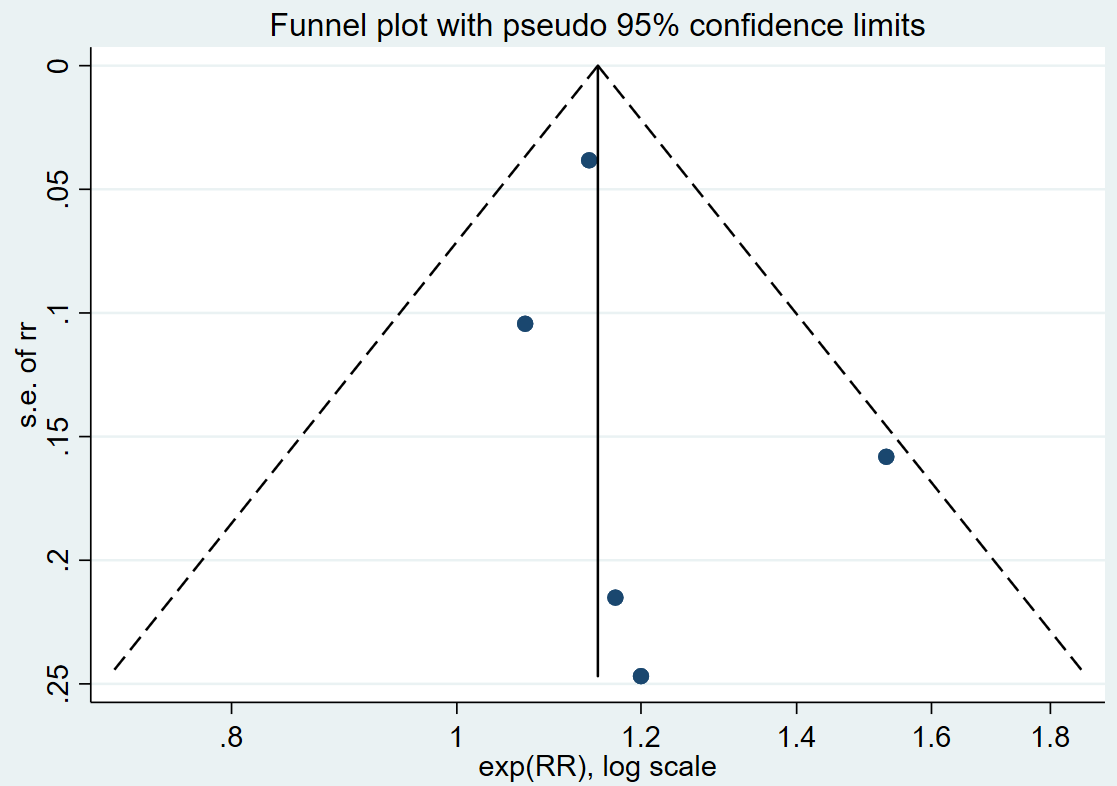

Supplement: Supplementary file 2 — Supplementary Material 2 [file 13690_2025_1659_MOESM2_ESM.docx]
